# Upper limb children action-observation training (UP-CAT): a randomised controlled trial in Hemiplegic Cerebral Palsy

**DOI:** 10.1186/1471-2377-11-80

**Published:** 2011-06-28

**Authors:** Giuseppina Sgandurra, Adriano Ferrari, Giuseppe Cossu, Andrea Guzzetta, Laura Biagi, Michela Tosetti, Leonardo Fogassi, Giovanni Cioni

**Affiliations:** 1 Scuola Superiore Sant'Anna, Piazza Martiri della Libertà, 33 - 56127 Pisa, Italy; 2 Department of Developmental Neuroscience, Fondazione IRCCS Stella Maris, Viale Del Tirreno, 331, 56128, Calambrone (Pisa), Italy; 3 Children Rehabilitation Unit, S.Maria Nuova Hospital, Viale Risorgimento, 80, 42123 Reggio Emilia, Italy; 4 Department of Neuroscience, University of Modena and Reggio Emilia, via Pietro Giardini, 1355 - loc. Baggiovara, 41126 Modena, Italy; 5 Department of Neuroscience, Section of Physiology, University of Parma, Via Volturno, 39 - 43125 Parma, Italy; 6 Division of Child Neurology and Psychiatry, University of Pisa, Via dei Giacinti, 2, 56128 Pisa, Italy

## Abstract

**Background:**

Rehabilitation for children with hemiplegic cerebral palsy (HCP) aimed to improve function of the impaired upper limb (UL) uses a wide range of intervention programs. A new rehabilitative approach, called Action-Observation Therapy, based on the recent discovery of mirror neurons, has been used in adult stroke but not in children. The purpose of the present study is to design a randomised controlled trial (RCT) for evaluating the efficacy of Action-Observation Therapy in improving UL activity in children with HCP.

**Methods/Design:**

The trial is designed according to CONSORT Statement. It is a randomised, evaluator-blinded, match-pair group trial. Children with HCP will be randomised within pairs to either experimental or control group. The experimental group will perform an Action-Observation Therapy, called UP-CAT (Upper Limb-Children Action-Observation Training) in which they will watch video sequences showing goal-directed actions, chosen according to children UL functional level, combined with motor training with their hemiplegic UL. The control group will perform the same tailored actions after watching computer games. A careful revision of psychometric properties of UL outcome measures for children with hemiplegia was performed. Assisting Hand Assessment was chosen as primary measure and, based on its calculation power, a sample size of 12 matched pairs was established. Moreover, Melbourne and ABILHAND-Kids were included as secondary measures. The time line of assessments will be T0 (in the week preceding the onset of the treatment), T1 and T2 (in the week after the end of the treatment and 8 weeks later, respectively). A further assessment will be performed at T3 (24 weeks after T1), to evaluate the retention of effects. In a subgroup of children enrolled in both groups functional Magnetic Resonance Imaging, exploring the mirror system and sensory-motor function, will be performed at T0, T1 and T2.

**Discussion:**

The paper aims to describe the methodology of a RCT for evaluating the efficacy of Action-Observation Therapy in improving UL activity in children with hemiplegia. This study will be the first to test this new type of treatment in childhood. The paper presents the theoretical background, study hypotheses, outcome measures and trial methodology.

**Trial Registration:**

NCT01016496

## Background

Cerebral palsy (CP) is "a group of disorders of the development of movement and posture causing activity limitation that are attributed to non-progressive disturbance that occurred in the developing foetal or infant brain. The motor disorders of CP are often accompanied by disturbance of sensation, cognition, communication, perception, and/or behaviour, and seizure disorder" [1]. Therefore, children with CP are faced with a variety of motor and sensory impairments that have an impact on their arm function [1,2]. It is the most common cause of physical disability in childhood, occurring between 2 and 3 per 1000 live births. Hemiplegic forms, characterised by a clinical pattern of unilateral motor and sensory impairment, constitute the most frequent expression of CP (more than 38% of cases) and the second in term of prevalence, after diplegia, in premature infants (around 20% of cases) [3-5]. Typically, the upper limb (UL) is more involved than the lower one, resulting in a significant reduction of the effective use of the arm and hand in the daily activities in terms of activity and participation limitations.

There are many models of intervention targeting deficits in UL function that aim to reduce activity limitations for children with hemiplegia. In a recent review [6] the authors identified four main interventions: intramuscular botulinum toxin A (BoNT-A) combined with upper-limb training; constraint-induced movement therapy (CIMT); hand-arm bimanual intensive training (HABIT) and neurodevelopmental therapy. They demonstrated that no one treatment approach seems to be superior; however, injections of BoNT-A provide a supplementary benefit to a variety of upper limb-training approaches. Both CIMT and HABIT rely on UL intensive training, either unimanual or bimanual, respectively. They are underpinned by theories of motor learning and neuroplasticity that describe a correlation between improved motor function and the use of "massive" or "repetitive" practice.

A possible new rehabilitative approach aimed to improve movement control of the UL is based on the discovery of mirror neurons (MNs) in the ventral premotor cortex (area F5) and in the inferior parietal lobule (area PFG) of the monkey. They constitute a class of visuomotor neurons discharging both when a monkey performs a goal-directed motor act (e.g. grasping an object) and when it observes another individual performing the same or a similar motor act [7-9].

A comparable Mirror system (MS) has been identified also in humans, using several techniques such as Positron Emission Tomography (PET), functional Magnetic Resonance Imaging (fMRI) and Trancranial Magnetic Stimulation (TMS). The two main nodes of human MS are the inferior parietal lobule (IPL) and the ventral premotor cortex (PMv), plus the caudal part of the inferior frontal gyrus (IFG) (see for review Rizzolatti et al 2009 [10]). This network transforms the sensory representations of observed motor acts into their motor representations. This discovery has radically changed the notion of well-separate neural substrates for sensory and motor processing by suggesting that perception and action are much more tightly linked than previously believed [11]. Note that the *mere observation *of motor acts performed by other individuals determines a clear increase in activity in the above-mentioned fronto-parietal neural network. Moreover, when TMS stimulation is delivered during action observation, it produces an increase in the excitability of the corticospinal pathway (i.e. increased amplitude of motor evoked potentials [MEPs]) and the recorded pattern of muscle activation is very similar to the pattern of muscle contraction recorded during the execution of the same action [12]. On the basis of the mechanism matching action observation with action execution, it has been postulated that the MS is the basis for action understanding and imitation. These functions make it a very good candidate for observational learning [11,13-15].

An important characteristic of MNs is that most of them discharge in association with specific motor acts (e.g., grasping, holding, tearing) rather than with the simple movements [8,9]. This goal-directed activity relies on the fact that MNs are basically motor neurons and it is well known that the premotor areas and, in part, even primary motor cortex (M1) encode the goal of a given motor act [16-19].

This concept has been recently further supported by two studies on premotor grasping neurons recorded in monkeys trained to use two types of pliers (normal and reversed) that allowed to obtain the same goal (grasping food) by performing opposite movements. In a first study it has been found that these neurons fire when the monkey uses both types of tools to grasp the food, showing that the activity is related to the goal of the motor act and not to the movements (fingers flexion or extension) the monkey used to achieve it [20]. Moreover, in a second study [21] it was demonstrated that after the training for the use of pliers, all neurons responding to the observation of hand grasping also responded to the observation of grasping with pliers and many of them also to the observation of another, different tool, to which the monkeys were not trained (spearing with a stick). These results showed that both tools were effective in triggering MNs, in spite of the fact that they markedly differed from one another (as well as from the hand, the natural grasping effector) both in their visual aspects and in their movement kinematics. In other words, it is plausible that, once a general set has been learned, a generalisation occurs to other implements, even to those the monkey has never used. This finding could have an important rehabilitative relevance, because by amplifying the tools the patients will enrich their use of UL.

Motor acts are normally combined to form goal-related actions. Interestingly, the same motor acts can be included in different actions having different ultimate goals. Recently, it has been shown that the visual responses of a subset of premotor and inferior parietal MNs, studied during both the observation and execution of grasping acts embedded in different actions (e.g., grasping to place or grasping to eat), are modulated by the final action goal (placing or eating) [22,23]. Altogether, these data indicate that during observation of an agent performing an action, the observer codes the final goal of the observed action (corresponding to the agent's intention) and the goal of the motor act included in that specific action. These two aspects are fundamental for a rehabilitation therapy based on action observation.

An important assumption for an observation-based therapy is the plasticity of the MS. Several studies [24-26] indicate a strong role of the MS in representing previously acquired motor skills. These fMRI studies examined the mirror activations in person expert in specific motor skills (dance) and compared them with the activations determined by the same stimuli in individuals having different motor experience. The results showed that the observed dance steps were mapped onto the observers' premotor-parietal motor system and that the activation was stronger in individuals expert in performing them. Thus, during observation, observers tend to 'resonate' more strongly with actions already embodied in their own motor repertoire.

These data prompt the hypothesis that if observation is accompanied by imitation one should expect a higher activation of the MS. In a fMRI study where non-guitarists were asked to imitate unfamiliar guitar chords (a task chosen to represent the initial stage of imitation learning) Buccino et al [27] demonstrated that the basic circuit underlying this capacity includes the MS that starts to be active during the observation of the guitar chords and it is permanently active also during motor preparation and execution. Moreover, the left dorsolateral prefrontal cortex (DLPFC, most likely area 46) was found activated mainly during motor preparation of imitative execution, suggesting its possible role in the selection and recombination of the individual motor elements, as represented in the MS, into a new motor pattern.

These findings were confirmed by a more recent study conducted by Vogt et al [28] showing that MS is more strongly involved during the observation of novel actions than of previously practised actions.

The possibility to use for neurological patients an observation/imitation approach in the Action-Observation Therapy has been exploited in two studies with hemiplegic adults after stroke. In the first clinical trial, Ertelt et al [29] enrolled 16 participants with moderate post-stroke hemiparesis who were randomly assigned to either the experimental or the control group. The experimental group performed an action-observation training based on the combination of action observation with repetitive motor training of the observed actions for 90 minutes per day with the paretic hand. Actions of increasing complexity were observed and imitated each day for 18 days. The control group performed the same UL actions of the experimental group on verbal instruction, while they observed only geometrical symbols and letters. Significant functional improvement on standard scales occurred for experimental group compared with controls and was maintained at 8 weeks post-training. In addition, before and after training, participants of both groups performed an independent object manipulation task while scanned with fMRI. The data revealed, only in the experimental group, a significant increase in neural activity of motor-related brain regions including the MS. More recently, Franceschini et al [30] performed an observational study, without control group, with twenty-eight chronic stroke patients with UL impairment. All patients underwent for four weeks, five days a weeks, a rehabilitation treatment based on observation of video-clips presenting hand daily actions, followed by the imitation of those same actions with the affected limb. In all function scales, scores improved significantly after treatment and changes were maintained also after two-months.

To date, there are no studies using action-observation model in children. In this project, we will propose, for the first time, a new paradigm of UL rehabilitation, called Upper Limb Action-Observation Training (UP-CAT), built as a randomised controlled trial (RCT) according to Consolidated Standards of Reporting Trials (CONSORT) Statement for Randomised Trials of non-pharmacologic treatment guidelines [31,32] to examine the efficacy of Observation To Imitate (OTI) in improving the UL activity in children with Hemiplegic Cerebral Palsy (HCP).

## Methods/Design

The following hypotheses will be tested by means of a RCT comparing the effects of UL action observation versus physical practice in improving the UL activity in children with HCP:

• If the MS works in terms of action goals, regardless of the kinematic properties, there will be a better improvement in the goal of UL use rather than in the kinematics of UL;

• OTI will be able to induce more changes in UL activity than those due only to the physical practice of the same actions;

• OTI will result in greater enhancement of daily activity performance;

• OTI will result in a greater mirror circuit re-organisation compared to action execution only and this cortical plasticity will be retained for longer periods.

These experimental hypotheses will address the following specific aims:

• OTI model is a novel training method where the main core is to observe an UL performing tailored goal actions, with the intent to imitate. This study will determine if this model is useful to enhance activity performance and, if there are improvements, for how long that effect will be retained;

• If OTI model will enhance the activity performance, new models of the rehabilitation setting in clinical practice will be proposed;

• If OTI model results in greater brain plasticity, the functioning of MS and its role in learning also in children with brain lesion will be demonstrated;

• If OTI model will be a good tool, it will guide practice also in other disabilities.

### Study design

A matched pairs randomised, evaluator-blinded trial will be carried out using an action-observation intensive training program to evaluate the efficacy of OTI compared to physical practice only in children with HCP aged 5 to 15 years. Assessment will be performed at baseline (T0, on the week preceding the onset of the treatment), and then on the week (T1) and 8 weeks (T2) after the end of the treatment. A further assessment will be performed at 24 weeks (T3) after the end of the treatment to evaluate the retention of effects.

**Figure 1:**
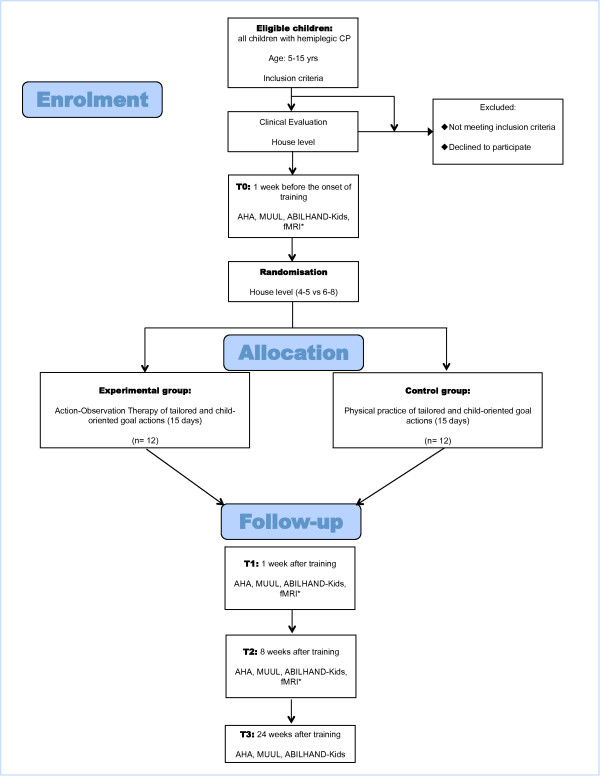
**Flow-chart of UP-CAT study according to CONSORT guidelines**. Abbreviations: AHA: Assisting Hand Assessment MUUL: Melbourne Assessment of Unilateral Upper Limb Function fMRI: functional Magnetic Resonance Imaging * only in a subgroup of children enrolled.

The experimental design and outcome measures are depicted in Figure 1.

### Study sample and recruitment

Potential participants will be identified according to strict criteria, listed below, from hemiplegic children' databases of the Department of Developmental Neuroscience of the IRCCS Stella Maris (Pisa, Italy) and of Unit of Children Rehabilitation of S. Maria Nuova Hospital (Reggio Emilia, Italy). Suitable children and their parents will then be invited to participate in the randomised trial and informed consent to participate will be obtained from the child and/or by her/his parents prior to enrolment in the RCT.

The trial has been approved by the Ethics Committee of both Institutions.

#### Inclusion criteria

This study will include children if they met the following criteria:

• confirmed diagnosis of HCP according to 2005 definition (MRI and clinical history) [33];

• aged 5 to 15 years at time of recruitment;

• predominant spasticity rather than dystonia interfering with UL function according to the classification of motor type by Sanger et al [34] with Modified Ashworth scale (MAS) grade ≤ 2 [35];

• mild or moderate severity of UL disability i.e. active use of affected UL from poor active assist use to complete spontaneous use according to House Functional Classification System [36,37] grade between 4 and 8;

• sufficient cooperation and cognitive understanding to participate in the activities;

• good attention;

• no sensory impairment;

• no history of seizures or seizures well controlled by therapy;

• children living near to one of the two clinical centres (Pisa or Reggio Emilia);

• parents able to commit to an intensive therapy program for 3 weeks.

For a subgroup of children performing fMRI further inclusion criteria will be i) sufficient cooperation to perform imaging studies for 45 minutes and ii) no exclusions for 1.5 Tesla Magnetic Resonance System such as no metal implants, no shunts.

#### Exclusion criteria

• moderate or severe muscle spasticity and/or contracture (MAS > 2) [35] which would require spasticity management or orthoses;

• activity level at House Functional Classification System [36,37] < 4;

• uncontrolled epilepsy;

• previous orthopaedic surgery in the UL;

• BoNT-A injection in the UL within 6 months prior to study entry.

### Sample size

According to CONSORT guidelines [31,32] the sample size estimates were based on projected treatment effect on the primary outcome measure, the Assisting Hand Assessment (AHA). The AHA scale's responsiveness to change has been shown in a study in which Eliasson et al [38] used the scale as the outcome measure in evaluating the effects of a modified model of CIMT. The authors reported a significant effect size of 1.16. Calculation by a statistician indicated that in order to detect a 1.16 effect size at significant level of 0.05 and 80% power a minimum sample size of 12 per group is required in our study.

### Randomisation

After enrolment, children will be block randomised into pairs according to their activity level at House Functional Classification System [36,37] (grade 6-8 vs 4 or 5) using a computer generated set of random numbers. A computer random allocation will also be used to randomly assign the subjects of each pair to experimental or control group by a computer random allocation. We choose a matched pairs design because it minimises the likelihood of group differences at baseline that has often been present in UL rehabilitation studies [39]. All randomisation, sequence generation, and preparation of group allocation materials will be performed by a third party, who has no direct contact with the clinical aspects of the trial. The master list of random numbers will be located in locked cabinets only accessible at completion of the RCT for analysis.

### Blinding

In order to make blind the study the children enrolled and their parents will be informed about general description of the design of the study, so that they know that there are two parts during the treatment, one of observation and one of action, but not the kind of observation. Outcome measures (Melbourne Assessment of Unilateral Upper Limb Function and AHA) will be videotaped, randomised and scored by assessors blind to group allocation and order of assessment. The treating therapists and study personnel, committed to help during the treatment, will be not blinded of group allocation. Outcome measures will be administered by a therapist blind of group assignment and scored by different assessors also blind to group allocation.

### Therapy protocols

The group of physical therapists and child neurologists planned 15 sets of daily life UL exercises. Each set is composed by three sequential UL goal actions of increasing complexity. The first 8 sets are unimanual exercises (total of 24), while the last 7 sets bimanual (total of 21) where the two upper limbs have different roles in an integrated action. In order to grade the activities with the range of children capabilities two series of sets were conceived, one for children grade from 6 to 8 at House Functional Classification System [36,37] and one for those grade 4 or 5. The setting of all exercises is the same but the proposed type of movement (i.e. range of movement) is simplified for the children with more severe impairment (grade 4 or 5). Each action of the two series performed by an actor was videotaped so that the videos showed only the hand and arm and in the first perspective; then each of this videos was edited so that each lasted 3 minutes. The actor used one or two hands respectively for the unimanual and bimanual exercises; collected videos were also tipped over, in order to have the same videos for the right and for the left hand. Moreover, 15 sets of computer games with no biological movements were chosen (i.e. quiz games, crossword, Simons etc.).

### Study treatment

The total dosage of intensive rehabilitation will be 15 hours. Each daily session will last 60 min with a total of one session for 15 consecutive working days. During the rehabilitation sessions, children will be sitting on a chair with their arms placed on a table; to provide a suitable freedom range of movement of upper limbs, the child's chair will be adjusted so that the table is at waist height. A large monitor screen (22 inches) will be positioned 1-m distance in front of them. A member of the staff will sit on the child's affected side in order to prevent the children from leaving the table, to assist them in maintaining attention on the video sequences or videogames and during the execution of the task. Another person of the staff will operate the setup (on/off of the monitor, use of keyboard, arrangement of the setting on the table).

**Figure 2:**
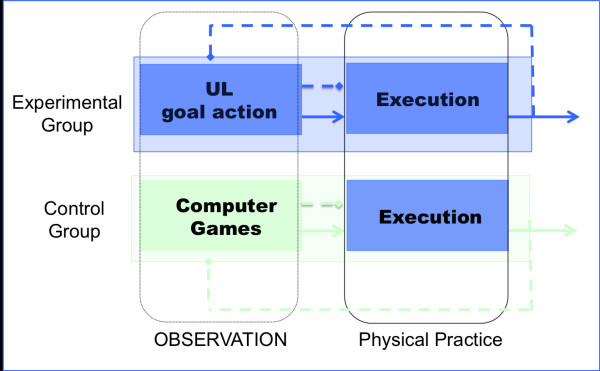
**Layout of Study Treatment**.

The children randomised in the experimental group ("action observation therapy") will watch the video sequence and afterwards the same objects shown on the video will be placed on the table and the children will be requested to perform the observed action for 3 min with their hemiplegic UL or both limbs, as demonstrated on the video. Each video sequence will be presented twice during the training (Figure 2).

Children randomised in the control group will match the design of experimental session except for the content of the videos showing computer games with no biological movements (Figure 2). Moreover, the children will play the games without using their hands, i.e. the person of the staff will use the keyboard to go on over the game. Following verbal instruction given by the staff, control group will carry out the same exercises in the exact order performed by the experimental group.

### Outcome measures

#### - Classification of the sample

The children entered into the study will be classified according to:

##### - House Functional Classification System

This is a reliable tool for assessing upper extremity function in children with CP [36,37]. It was developed for the evaluation of function in the affected hand after surgery for thumb-in-palm deformity in children with spastic hemiplegic CP [36] and has been used to evaluate children before and after upper extremity BoNT-A injections [40]. Even if it was constructed for hemiplegic CP, in some studies it is used for each hand separately in all types of CP through observation of the child in activities requiring bimanual hand function [41]. The classification consists of 9 grades ranging from a hand that is not used at all (grade 0) to one that is used spontaneously and independently from the other hand (grade 8). The House has been reported to have an excellent interrater (Intraclass Correlation Coefficient, ICC = 0.92) and intrarater reliability (ICC = 0.94) [37].

#### - Primary and secondary outcome measures

On the basis of our scientific hypothesis, the primary outcome measure of interest is bimanual UL activity performance using the AHA. Secondary measures include measure of unimanual capacity (Melbourne Assessment of Unilateral Upper Limb Function, MUUL) and the bimanual daily activities at home and in the community (ABILHAND-Kids). At the beginning of the study we intended to use also the Jebsen-Taylor Hand Function Test [42], but we realised that the test could be frustrating for some children with more severe impairment and, since the measure of dexterity was not an aim of our proposed rehabilitative approach we decided to skip this test from our measures.

##### - Assisting Hand Assessment (AHA)

The primary outcome was the Assisting Hand Assessment (AHA, version 4.4) that is a standardised, performance-based test for use with children aged 18 months to 12 years, who have an unilateral upper limb impairment. It evaluates the spontaneous use of the assisting hand during a semi-structured 10-15 minutes play session with specific toys (from the AHA test kit) requiring bimanual handling [43,44]. The play context is age appropriate so there is one context for children 18 months to 5 years (Small kids AHA) and others (two different board games) for children aged 6-12 years (School Kids AHA). The forms are directly comparable as demonstrated by a test-retest of alternate forms of AHA [45]. The AHA is videotaped in a standardised manner and the subsequent scoring procedures produce a raw sum score ranging from 22 (low ability) to 88 (high ability) and a logit measure from -10.18 to +8.70. The AHA can only be administered and scored by a certified rater. Interrater, intrarater and test retest reliability of the AHA have a high ICC [45,46]. The Smallest Detectable Difference (SDD) over time indicated that a change in School Kids AHA scores from one test session to the next must be 3.65 sum scores (0.76 logits) or more to be considered a true change with 95% probability [45]. Also, the scale responsiveness to change was demonstrated in intervention studies focusing on forced use therapy [47], CIMT [38,44,46,48,49], HABIT [50] and, recently, modified CIMT followed by task-specific training of goal-directed bimanual play and self-care activities [51,52].

##### - Melbourne Assessment of Unilateral Upper Limb Function (MUUL)

The MUUL is an evaluative tool that measures unilateral upper extremity quality of movement in children with neurological impairments aged from 5 to 15 years [53,54]. A modified MUUL for children in the age range of 2 to 4 years has recently been developed [55]. MUUL is a criterion-referenced test based on 16 items scored on a 3- to 5-point ordinal scale comprising tasks that are representative of the most important components of unilateral UL function (reach, grasp, release, and manipulation). Most items are further subdivided in 2 to 4 sub-items (total of 37 sub-items) that represent an aspect of the required movement, such as range of movement, fluency, target accuracy, speed, and quality of movement. The total score can range from 0 to 122 points and can be converted to a percentage. According to International Classification of Functioning, Disability and Health (ICF) the MUUL measures both capacity in the domain of activity and some aspects at the body functional level (i.e. range of motion, fluency) [46]. The test is administered with the child's performance recorded on videotape for subsequent scoring. The interrater reliability for the total score is 0.97 (ICC). Percentage of agreement of 32 sub-items varied between 35% and 95%. The intrarater reliability is also high (ICC 0.97) [56]. In the first reliability study of MUUL, Randall et al [53] demonstrated that when two assessments of the same child scored by the same therapist differ by more than 14.3 points (12%) it is probable that it reflects a true change in function rather than an error in measurement. Recently, Klingels et al [55] demonstrated that the SDD is 8.99% instead of 12%. The MUUL has been used in several intervention studies [57-59] but some studies have suggested that the MUUL might not be sufficiently sensitive to changes brought about by BoNT-A treatment because the SDD was smaller than recommended. Therefore, further investigations were warranted [40].

##### - ABILHAND-Kids

The ABILHAND-Kids is a short questionnaire that measures 21 mainly bimanual daily activities referred to the activity domain of the ICF. The difficulty experienced by the child to perform the required tasks is scored by a parent on a 3-point ordinal scale (impossible, difficult, easy). It has been validated and calibrated in children with CP (age 6-15) and has a high reliability (R = 0.94) and a good reproducibility over time (R = 0.91). The questionnaire was developed using the Rasch measurement model which provides a method to convert the raw scores into a linear measure located on a unidimensional scale [60]. In a recent study where a modified CIMT followed by task-specific training of goal-directed bimanual play and self-care activities were used in children with HCP a high effect size (Cohen's d: 1.01) was reported [46,51,52].

### Brain Reorganization

Some of us [61] recently have performed a study using fMRI to explore the activation of human anterior intraparietal area (AIP) during the observation of complex object-manipulation tasks (e.g. inserting a key in a lock and turning it) as compared to simple tasks (e.g. whole hand grasping of an object) executed with the left and the right hand in a first person perspective. The results showed that, in general, both complex and simple tasks produced an activation of the fronto-parietal mirror system and that the activity of AIP in each hemisphere was higher during observation of the contralateral hand (hand identity effect). A Region-Of-Interest (ROI) analysis of the parietal activations responding to hand identity showed that each AIP was more active during the observation of complex with respect to simple tasks. In the right AIP this effect was stronger during observation of the contralateral hand, in the left AIP was stronger during observation of both hands. This complexity related property was not observed in the other activated areas. These findings support the concept that the observation of motor acts retrieves the internal representation of those same acts in the observer's motor system (direct-matching hypothesis based on the mirror neuron mechanism). This study was conducted in adults, but our group have large experience in performing fMRI with children [62-67]. To obtain a better compliance by the children the paradigm used with the adults was reduced in the time of acquisition and only two functional series (see later) will be acquired.

The main aims of this part of the study will be:

• to explore the MS in children with HCP and

• to provide, if there are, some evidences of plastic changes after action-observation training either on the MS or in the somatosensory cortex.

To verify the first hypothesis, a group of healthy age-matched children will be enrolled. For the second hypothesis, a subgroup of children with HCP recruited for the study and randomised to the experimental or control group (see inclusion criteria) will perform fMRI before (T0) and after the training (T1 and T2) (see Figure 1).

#### - fMRI: experimental design

From the activation methods of our previous study [61], a set of 12 video-clips showing object manipulation, lasting 8 seconds each, will be used in this experiment: 3 complex actions and 3 simple actions, performed with the right or the left hand. The observed hand will be presented in a first person perspective. The complex actions are: i) grasping little cubes to put into a box, ii) performing a simple scale on a piano keyboard, iii) grasping a key, putting it into a lock and turning it. The simple actions consisted in a whole hand grasping of a small box in the same visual context as the corresponding complex actions. The two sets (simple and complex) of clips are perfectly balanced as to the visual content (luminance, amount of visual information). The arm trajectory is the same for both simple and complex actions. The observation of the static initial frame of each clip is used as control condition. The experiment will be performed using a block design format and it will consist of 2 functional series containing all the three types of action ("mixed" condition). In each functional series, four conditions are codified: simple tasks performed by the right hand (SR), simple tasks performed by the left hand (SL), complex tasks performed by the right hand (CR), complex tasks performed by the left hand (CL). Each series presents two blocks for each condition, in random order and in a counterbalanced manner, interleaved with as many control blocks. For each functional series each block consists in the presentation in random succession of the three different types of action corresponding to the same condition. The stimuli will be presented binocularly, displayed on LCD goggles (VisuaStim XGA - Resonance Technology, USA). Children will be instructed to observe the video-clips, maintaining fixation in the middle of the screen. The maintenance of the fixation will be confirmed by continuously monitoring gaze through an infrared camera, mounted inside the goggles (sample frequency 60 Hz). Moreover, in the same MRI session, children will perform also another fMRI study using a sensory-motor task, alternating sensory stimuli and motor stimuli; the former consisting in the palm and fingers passively brushed by an external operator by means of a wooden spatula, at a frequency of about 1 Hz, while in the motor task, we will ask to the children sequentially moved all the fingers in opposition to the thumb ("open and close your hand"). One series for each hand will be acquired. We will start with the unimpaired hand to check that the children have understood the task and that they remember it. The subjects will be asked to keep their eyes closed; ambient scanner noise will be constant throughout baseline and activation periods, and attenuated by occlusive ears plugs.

#### - fMRI: Image acquisition, processing and analysis

Blood oxygen level dependent (BOLD) responses will be acquired by a 1.5 T MR system (General Electric Signa Horizon LX Milwaukee, USA), equipped with echo-speed gradient coil and amplifier hardware. Activation images will be acquired using Echo Planar Imaging (EPI) gradient-recalled echo sequence (TR/TE/flip angle = 3000 ms/50 ms/90°, FOV = 240 × 240 mm, matrix = 64 × 64, 5 mm thick slices). For MS exploration, time-course series of 132 scans for each volume will be collected in 16 blocks alternating between control and action conditions, resulting in an acquisition time for each series of 6'36'', repeated 2 times. For motor-sensory tasks, time curse series of 100 scans for each hand will be collected in 16 blocks alternating between Motor and Sensory conditions (each followed by a period of Rest), resulting in an acquisition time for each series of 5'00''. Each first block of each series included 4 initial extra scans to allow the stabilization of signal and this period will be eliminated from subsequent analysis. A volumetric set of data (3D FSPGR: TR/TE/TI/flip angle = 21.1 ms/3.8 ms/700 ms/10°; FOV = 280 × 280 mm, matrix = 256 × 256) will be also acquired to generate a 3D whole brain reconstruction. Data analysis will be performed using the Brain Voyager QX software package (Brain Innovation, Maastricht, the Netherlands). Before statistical analysis, raw data will be corrected for head movements and high-temporal filtered in order to exclude temporal drifts. For each subject, all the two-dimensional functional data will be co-registered, concatenated, aligned to the three-dimensional high-resolution images and finally transformed into Talairach space [68]. A General Linear Model (GLM) approach will be used to generate statistical parametric maps, using a hemodynamic response function modelled on the standard Boynton's function [69] and considering the four conditions: SR, SL, CR, CL. Moreover, because the side of HCP in the children enrolled could be right or left, to correct this effect, we will treat the processing delivered by the dominant hand as the unimpaired hand and whose by the non dominant hand as the impaired hand. A ROI analysis will be conducted on the statistically significant clusters coming out from the application of the GLM analysis. In these ROIs, a single-subject analysis will be also performed measuring the average per cent signal change of each individual subject.

To test our experimental hypotheses we plan these main analyses, as follows:

• to investigate whether the used stimuli is able to activate the MS in children with HCP, we will contrast activity for all the observed actions vs the static conditions (hand action observation, all stimuli > static conditions) and if the activity in children with HCP will be different with respect to normal functional response (healthy children);

• to explore whether there are areas differentially responding to the identity of the observed hand, we will compare the activity related to observation of the simple and complex tasks performed by the corresponding impaired (non-dominant) hand with those performed by the unimpaired (dominant) hand;

• to study whether there are some changes after UP-CAT, a group analysis of intra-participant change in activation will be undertaken using a ROI approach both for MS and for the sensory-motor task.

### Analyses

Clinical Data will be managed and analysed using the Statistical Package for Social Sciences (SPSS version 16.0). Descriptive statistics (means, standard deviation) will be calculated to summarise the data set for both groups and to identify potential baseline differences between the groups; p values will be used to indicate the strength of the evidence, a significant level of 0.05 will be used. Initially between group differences will be evaluated at baseline (T0) assessment point using Mann-Whitney U independent sample test for all Outcome Measure chosen. Within group changes between baseline and follow-up assessment (T1 and T2) will be evaluated using Wilcoxon matched-pairs sign rank test. A second level analysis with independent sample t test of the differences between the follow-up assessment and baseline (T1 vs T0 and T2 vs T0) will be performed for the comparison of the rehabilitative gain between the two groups. Moreover, Cohen's d values and their 95% confidence interval will be calculated to obtain a pre-post intervention effect size [70]. According to Cohen's work values of effect size below 0.2 will be considered to reflect "no effect", values from 0.2 to 0.5 a "small effect", values between 0.5 and 0.8 a "medium-sized effect", and values > 0.8 "large effect".

## Discussion

The paper presents the background and the design for a matched pairs randomised trial for evaluating the efficacy of action-observation therapy in improving UL activity in children with HCP. The study is the first to provide this new type of treatment in childhood. Furthermore, it is based on rigorous scientific designing, according to CONSORT guideline [33,34], as recommended by the evidence-based medicine. Finally, the set of exercises designed for the trial are child-oriented and tailored according to the grade of UL impairment.

## List of abbreviations

HCP: Hemiplegic Cerebral Palsy; CP: Cerebral Palsy; UL: Upper Limb; UP-CAT: Upper Limb Children Action-Observation Training; RCT: Randomised controlled trial; CONSORT: Consolidated Standards of Reporting Trials; BoNT-A: Botulinum toxin A; CIMT: constraint-induced movement therapy; HABIT: hand-arm bimanual intensive training; MNs: mirror neurons; MS: Mirror neuron System; PET: Positron Emission Tomography; MRI: Magnetic Resonance Imaging; fMRI: functional Magnetic Resonance Imaging; TMS: Trancranial Magnetic Stimulation; IPL: Inferior Parietal Lobule; PMv: ventral Pre-Motor cortex; IFG: Inferior Frontal Gyrus; MEPs: Motor Evoked Potentials; M1: primary motor cortex; DLPFC: dorsolateral prefrontal cortex; OTI: Observation To Imitate; MAS: Modified Ashworth Scale; AHA: Assisting Hand Assessment; ICC: Intraclass Correlation Coefficient; MUUL: Melbourne Assessment of Unilateral Upper Limb Function; SDD: Smallest Detectable Difference; ICF: International Classification of Functioning, Disability and Health; AIP: anterior intraparietal area; ROI: Region-Of-Interest; SR: Simple task Right hand; SL: Simple task Left hand; CR: Complex task Right hand; CL: Complex task Left hand; BOLD: Blood oxygen level dependent; EPI: Echo Planar Imaging; GLM: General Linear Model; SPSS: Statistical Package for Social Sciences.

## Competing interests

The authors declare that they have no competing interests.

## Authors' contributions

GCI and AF are the chief investigators and together with GS, LF and GCO designed and established this research study. GS, GCI and AF were responsible for the particular therapy contents and LF and GCO verify these contents according mirror neurons knowledge. GCI, GS and AF were in charge for subject recruitment, data collection, implementation of the studies in Pisa (GCI, GS) and Reggio Emilia (AF). MT, LB and AG were responsible for the design, implementation, data collection, analysis of the Advanced Brain Imaging studies. GCI, GS and AF will take lead roles on preparation of publications on the clinical outcomes of the study and AG, LF, GCI, MT and GCO will take lead roles on the neuroscience publications from the study. All authors have read and approved the final manuscript.

## Authors' information

Giuseppina Sgandurra is specialist in child neuropsychiatry and phD student in neuroscience at Scuola Superiore Sant'Anna, Pisa. Adriano Ferrari is specialist in neurology and rehabilitation medicine, professor of rehabilitation medicine at University of Modena and Reggio Emilia and head of Children Rehabilitation Unit, S. Maria Nuova Hospital, Reggio Emilia. Giuseppe Cossu is specialist in child neuropsychiatry and professor of child neuropsychiatry at University of Parma. Andrea Guzzetta is specialist in child neuropsychiatry and is researcher at Fondazione IRCCS Stella Maris in Pisa, which is a research hospital for child and adolescent with neurologic and psychiatric disorders, located in Calambrone, Pisa. Laura Biagi is medical physicist researcher at MRI Lab of Fondazione IRCCS Stella Maris. Michela Tosetti is medical physicist, head of MRI Lab at Fondazione IRCCS Stella Maris. Leonardo Fogassi is professor of psychophysiology at University of Parma. Giovanni Cioni is specialist in child neuropsychiatry, professor of child neuropsychiatry at University of Pisa and head of Department of Developmental Neuroscience, Fondazione IRCCS Stella Maris.

## Pre-publication history

The pre-publication history for this paper can be accessed here:

http://www.biomedcentral.com/1471-2377/11/80/prepub
